# Pseudoaneurysm of the Medial Superior Genicular Artery after Arthroscopic Partial Meniscectomy

**DOI:** 10.4055/cios.2009.1.3.173

**Published:** 2009-08-17

**Authors:** Kee Byoung Lee, Si Young Song, Duck Joo Kwon, Jun Shin, Sang Hoon Paik

**Affiliations:** Department of Orthopedic Surgery, Hallym University Sacred Heart Hospital, Anyang, Korea.

**Keywords:** Knee, Arthroscopy, Genicular artery, Psuedoaneurysm, Embolization

## Abstract

We describe a case of 43-year-old man who had a pseudoaneurysm of the medial superior genicular artery after arthroscopic partial meniscectomy with standard anterolateral and anteromedial portals. Pseudoaneurysm of the medial superior genicular artery has been reported at the previous superomedial portal site after arthroscopy. Described herein is a unique case that involved the medial superior genicular artery at the previous anteromedial portal site after arthroscopy. The pseudoaneurysm was successfully treated with transcatheter embolization.

Arthroscopic treatment of the knee has been considered a relatively low-risk procedure with a low complication rate. Vascular injuries are quite rare and represent 0.54% of all complications.1) The popliteal artery is the most vulnerable to damage during arthroscopy.^2^,3) Pseudoaneurysm of the medial superior genicular artery has been reported at the previous superomedial portal site after arthroscopy.4) To our knowledge, described here is the only case of a pseudoaneurysm that involved the medial superior genicular artery at the previous anteromedial portal site after arthroscopy.

## CASE REPORT

**Fig. 1:**
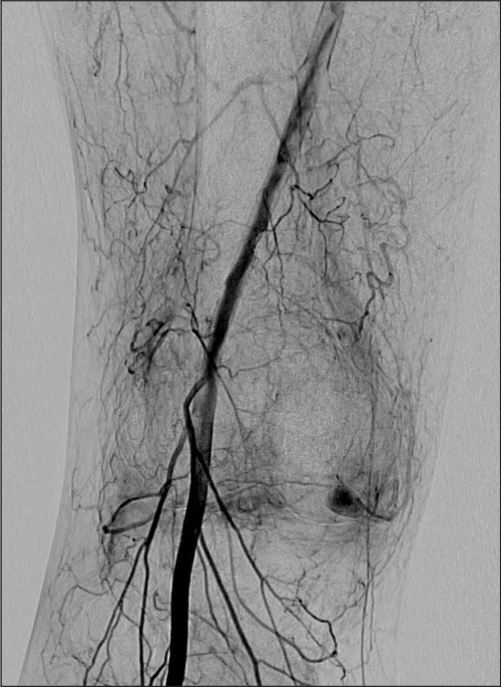
Arteriogram before embolization, showing a pseudoaneurysm of medical superior genicular artery.

**Fig. 2:**
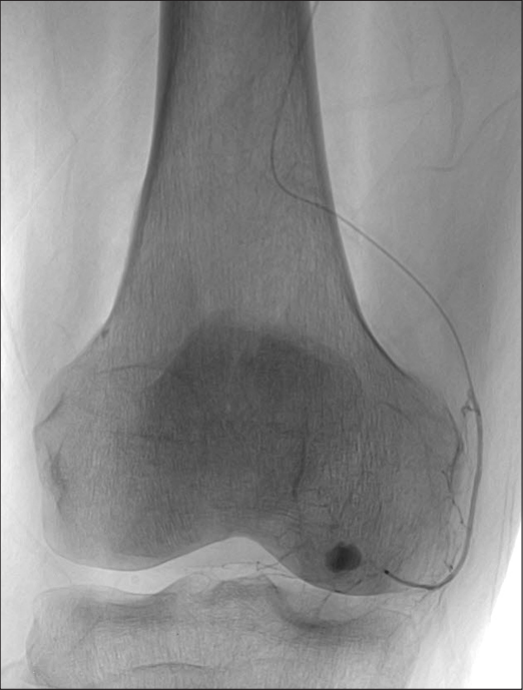
Embolization of the pseudoaneurysm with microcatheter.

**Fig. 3:**
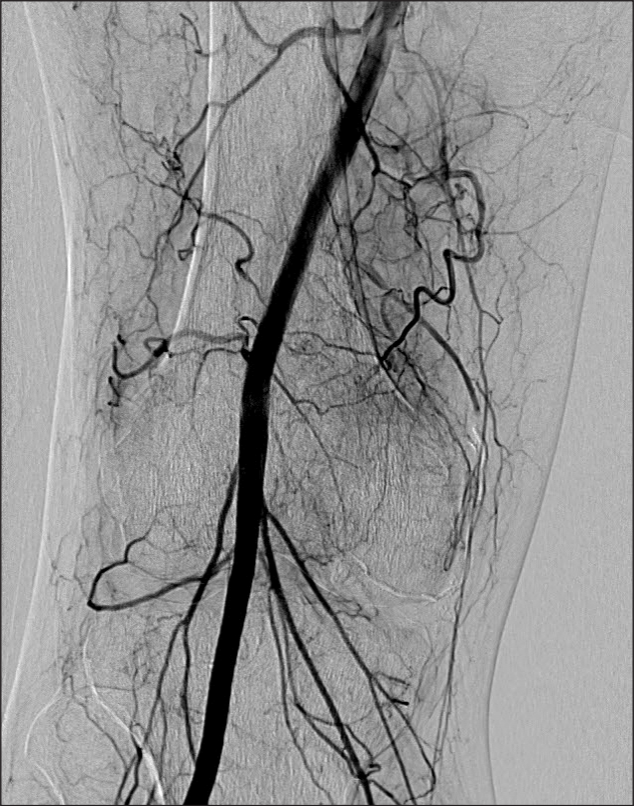
Arteriogram after embolization.

A-43-year-old man presented to us with a 6-month history of right knee pain. Physical examination revealed tenderness at the medial joint line and a mild effusion. Magnetic resonance imaging (MRI) examination revealed a tear of the medial meniscus. Diagnostic arthroscopy with standard anterolateral and anteromedial portals demonstrated a horizontal tear of the medial meniscus. A partial meniscectomy was done with the use of basket forceps through the anteromedial portal. The postoperative course was unremarkable and he was discharged on the 5th postoperative day. Eight days after the arthroscopy, a painful effusion was noted. This was aspirated and 40 ml of blood was obtained. At an outpatient clinic 3 weeks later, there were a persistent pain and a hemarthrosis. He complained of a pulsating mass in the region of the anteromedial portal site that developed 2 weeks previously. With referral to a radiologist, arteriography showed a 1.5 cm-sized pseudoaneurysm of the medial superior genicular artery (Fig. 1). The feeding arterial branch was superselected with a microcatheter (Fig. 2) and glue embolization was performed successfully. At the postembolization angiography, there was no evidence of a pseudoanerysm (Fig. 3). At the last follow-up 6 weeks after embolization, the patient was free of pain with no further vascular problem.

## DISCUSSION

Pseudoaneurysm after knee arthroscopy is very rare complication. The Complications Committee of the Arthroscopy Association of North America reported only 12 vascular injuries in 375,069 knee arthroscopies.1) Small5) reported there were no cases in 8,791 arthroscopic procedures performed by the most experienced arthroscopists in the United States. The case that is reported herein is the only iatrogenic pseudoaneurysm that was encountered by the senior one of us (K.B.L.) after more than 6,000 recorded arthroscopies between June 1988 and May 2007 (unpublished data).

Most pseudoaneurysms have involved the popliteal arteries after knee arthroscopy.^2^,3) Published reports suggest that the affected arteries are the lateral inferior genicular artery,^3^,6) the descending genicular artery,^3^,7) and the recurrent anterior tibial artery.4) Pseudoaneurysm of the medial superior genicular artery has been reported at the previous superomedial portal site after arthroscopy.4) An extensive review of the literature failed to reveal any description of a pseudoaneurysm of the medial superior genicular artery near the site of the anteromedial portal.

Clinically, the superomedial portal is not used as the standard portal and can be recommended only for a complete synovectomy. The anteromedial portal is most commonly used as an instrument portal and as an additional viewing portal. The high medial portal is also useful in direct access to the posterior horn of the medial meniscus and posterior cruciate ligament reconstruction. The frequent use of this portal probably results in injuries to the medial superior genicular artery.

A pseudoaneurysm is believed to develop following partial arterial laceration. The partial arterial laceration allows hemorrhage into surrounding soft tissues that confine it. An encapsulated hematoma is formed and endothelialization of the central cavity, which communicates with the arterial defect, forms the pseudoaneurysm.8) Complete arterial disruption would result in vascular constriction with little bleeding. Because of the small lumen, a partial disruption in the arteral wall is very rare.^3^,6)

Mariani et al.2) reported anatomic arterial variations and joint distraction may be risk factors for a pseudoaneurysm. In our opinion, an incision which is too small can induce a partial section of the artery and it will be necessary to force the sheath into the joint cavity, increasing the risk of an arterial injury. We think that an adequate stab incision and the careful use of a powered suction instrument will decrease the risk of a partial arterial laceration. In our case, the high position of the anteromedial portal and the inadequate stab incision may have contributed to this iatrogenic complication.

The symptoms of pseudoaneurysms of genicular arteries are hemarthrosis and /or a pulsatile mass.4) The diagnosis of a pseudoaneurysm should begin with a careful history and physical examination. Pseudoaneurysms should be considered in any patient with a recurrent hemarthrosis and a pulsatile mass after arthroscopy. Currently, the popularity of arthroscopic procedures with multiple portals may contribute to the higher incidence of vascular injuries. Although very rare, surgeons must keep in mind the possibility of a pseudoaneurysm.
